# Targeting the Nucleosome Acidic Patch by Viral Proteins: Two Birds with One Stone?

**DOI:** 10.1128/mbio.01733-21

**Published:** 2022-03-28

**Authors:** Floriane Lagadec, Vincent Parissi, Paul Lesbats

**Affiliations:** a Mobility of Pathogenic Genomes and Chromatin Dynamics, Fundamental Microbiology and Pathogenicity (MFP), CNRS UMR5234, University of Bordeaux, France; National Institute of Diabetes and Digestive and Kidney Diseases; Albert Einstein College of Medicine

**Keywords:** Prototype Foamy Virus, Kaposi's sarcoma-associated herpesvirus, cytomegalovirus, Gag, LANA, IE1, Retrovirus, Herpesvirus, Chromatin, Nucleosome

## Abstract

The past decade illuminated the H2A-H2B acidic patch as a cornerstone for both nucleosome recognition and chromatin structure regulation. Higher-order folding of chromatin arrays is mediated by interactions of histone H4 tail with an adjacent nucleosome acidic patch. Dynamic chromatin folding ensures a proper regulation of nuclear functions fundamental to cellular homeostasis. Many cellular factors have been shown to act on chromatin by tethering nucleosomes via an arginine anchor binding to the acidic patch. This tethering mechanism has also been described for several viral proteins. In this minireview, we will discuss the structural basis for acidic patch engagement by viral proteins and the implications during respective viral infections. We will also discuss a model in which acidic patch occupancy by these non-self viral proteins alters the local chromatin state by preventing H4 tail-mediated higher-order chromatin folding.

## INTRODUCTION

**FIG 1:**
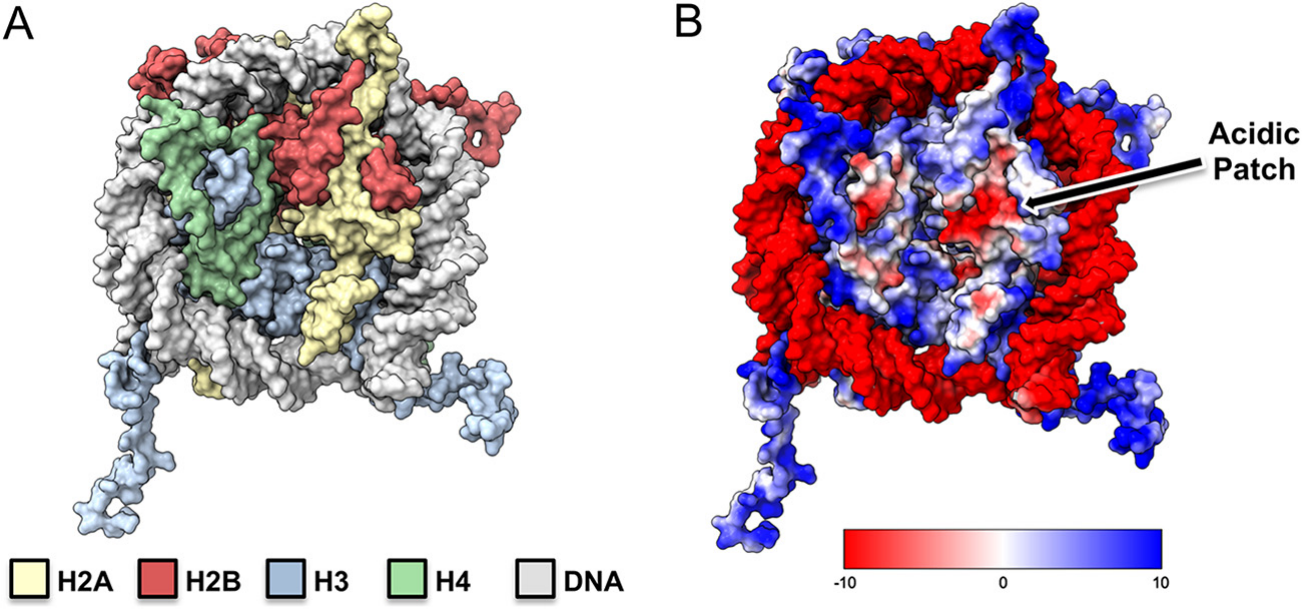
Nucleosome core particle and acidic patch. (A) Surface representation of the nucleosome core. Histones H2A, H2B, H3, and H4 are colored yellow, red, blue, and green, respectively, with DNA in light gray. (B) Electrostatic (coulombic) surface representation with H2A-H2B acidic patch indicated. Potentials were calculated using ChimeraX.

The DNA of eukaryotic genomes is tightly packed in a nucleoprotein structure called chromatin. The subunit of chromatin, the nucleosome, is composed of approximately 146 bp of DNA wrapped around an octamer of protein histones (H2A, H2B, H3, and H4) (Fig. 1A) (1). Compaction or relaxation of an array of nucleosomes generates a variety of chromatin assemblies that will be more or less permissive to different nuclear functions, like transcription, replication, and DNA repair (2, 3). The interaction between the H4 N-terminal tail and a charged contoured surface of H2A-H2B, called the acidic patch, has been shown to play a central role in the modulation of higher-order chromatin structures (4, 5). A tight regulation of these chromatin assemblies is crucial to maintain balanced cellular functions and avoid developmental diseases and cancers (6, 7).

To maintain cellular homeostasis, many proteins operate on chromatin, requiring a timely engagement of the nucleosome (5). The recent surge of cryoelectron microscopy (cryo-EM) structures revealed key molecular determinants for nucleosome recognition (8, 9). First, chromatin factors often make multivalent interactions with the nucleosome (core histones, histone tails, or DNA). Second, the H2A-H2B acidic patch serves as a hot spot for nucleosome binding. Third, many chromatin proteins use a conserved arginine residue, called arginine anchor, to associate with the acidic patch. Modulations on the accessibility of any of these three layers of interaction with the nucleosome offer leverages to finely tune the activities of the cellular proteins on chromatin.

However, nucleosome and acidic patch engagement is not restricted to cellular proteins. Penetrating the nuclear milieu is a mandatory step for many viruses, as they often rely on nuclear transactions to fulfill their replication cycle (10). However, as the nuclear envelope breaks down during cell division, maintenance of the viral genome in the nucleus can be compromised (11). To ensure adequate delivery and conservation of the viral genome during mitosis, viruses evolved strategies to tether their genome to the host chromatin. These include direct or indirect binding to genomic DNA or histone components (11, 12). Structural details for the direct engagement of the nucleosome are available for four viral proteins: Kaposi’s sarcoma-associated herpesvirus (KSHV) latency-associated nuclear antigen (LANA) protein, human cytomegalovirus (hCMV) immediate-early 1 (IE1) protein, prototype foamy virus (PFV) group antigen (Gag) protein, and integration complex (intasome). Except for the latter, all of them involve a conserved arginine anchor binding the acidic patch.

As a common interface crucial for chromatin structure and function, occupancy of the acidic patch by a foreign protein could compete with the H4 N-terminal tail and affect higher-order chromatin structure. In this minireview, we will discuss the structural basis for nucleosome acidic patch tethering by viral proteins and discuss a model in which, while maintaining their genome in the nucleus, the viruses hijack the acidic patch to remodel the intrinsic nucleosome-nucleosome dynamics, leading to an altered chromatin state beneficial for their replication.

## THE NUCLEOSOME ACIDIC PATCH

The fundamental unit of chromatin, the nucleosome, is a nucleoprotein complex of multiple functions (5). First, it allows the first layer of genomic compaction by wrapping around 146 bp of DNA. Second, nucleosomes can self-assemble into higher-order chromatin arrays to provide further degree of compaction of the genome. Third, the nucleosome acts as a signaling platform for chromatin transactions by offering a binding scaffold for chromatin factors (13). These interactions are fundamental in the regulation of the global chromatin architecture, which in turn modulates DNA processes.

The nucleosome offers a variety of binding surfaces: the central octameric histone core, the highly basic and flexible histone tails, and the exposed wrapped DNA (5, 9). The central histone core presents the largest surface area with a notable acidic patch: a cluster of negatively charged residues E56, E61, E64, D90, E91, and E92 of H2A as well as E102 and E110 of H2B (Fig. 1B). The acidic patch forms a groove where the acidic residues project within the pocket, forming a hydrophobic surface.

The recent plethora of nucleosome complex structures solved by cryo-EM provided a compelling source of information on the structural basis governing nucleosome recognition (9). These studies illuminated the central role of the acidic patch as a hot spot for chromatin factors. They also revealed that acidic patch binding commonly involves an arginine anchor motif. The canonical arginine anchor projects into the deeper acidic patch pocket comprised of the H2A acidic triad E61, D90, and E92. Careful examination of the landscape of the H2A-H2B acidic patch revealed additional binding zones that could accommodate variant arginine anchors as well as distinct chromatin factor-specific residues (9, 14). In addition to these high-resolution structures of nucleosome-bound protein complexes, a large-scale proteomic screen of nucleosome interacting factors was recently described and showed that more than 50% of nucleosome interactions are mediated by the acidic patch (15). These data confirm the importance of the acidic patch as a major hot spot for nucleosome recognition.

Chromatin is a dynamic structure subject to high structural plasticity. One current concept in chromatin dynamics is that an array of nucleosomes separated by linker DNA can fold into a more condensed structure called chromatin fiber (3). Several general factors influence the formation of higher-order chromatin folding (16). Among these, the dominant internucleosome interaction mechanism is driven by the interaction between the H4 tail and the acidic patch of a neighboring nucleosome (4, 17, 18). The H4 positively charged residues 16 to 23 mediate the internucleosomal interaction with the acidic patch and are required for higher-order chromatin organization. Cryo-EM structure of 30-nm chromatin fibers reconstituted in the presence of linker H1 provided important insights on the mechanisms of higher-order chromatin fiber folding (19). The structure reveals the molecular details of internucleosomal interactions mediated by H4 tail and the H2A-H2B acidic patch. However, the physiological relevance of the 30-nm fiber is still under debate, as several groups failed to observe such a structure *in cellulo* (20, 21). Although it cannot be ruled out that 30-nm chromatin fibers might exist under specific cellular contexts, several pieces of data suggest a long-range interdigitation model of nucleosome fiber folding (22). (For recent reviews on chromatin fiber see references 3 and 23). Additionally, some studies underline the central role of H4-tail/acidic patch in short-range and long-range nucleosome interaction (24, 25). Overall, it becomes clear that acidic patch occlusion by chromatin factors is incompatible with H4 tail-mediated internucleosome interactions. The functional implications underlying nucleosome-nucleosome destabilization that could result from the acidic patch competition are currently unclear. However, a hypothesis can be made from a recent study elucidating the structural basis for nucleosome binding by pioneer transcription factors. Pioneer transcription factors are specific transcription factors that bind and induce gene expression within condensed chromatin. Cramer and colleagues showed that binding of pioneer factor SOX to nucleosomal DNA induces a displacement of H4 tail incompatible with the formation of the canonical internucleosome array (26). The authors proposed that this H4 repositioning could destabilize chromatin packing and initiate its opening, thereby facilitating chromatin remodeling and transcription.

The balance between ground state (H4 tail-acidic patch-mediated nucleosome-nucleosome contacts) and protein-bound state (competition for the acidic patch) offers the possibility of modulating genomic architecture and fine-tuning chromatin transactions. Conversely, imbalance in acidic patch occupancy is a driver of diseases and cancer (6, 7).

## THE NUCLEOSOME ACIDIC PATCH AS A DOCKING STATION FOR VIRUSES

During viral infections, cellular homeostasis is disturbed. Viral proteins hijack cellular pathways and factors to reprogram the cell into a virus-producing factory. For some viruses, the nuclear compartment represents a mandatory step (10). However, delivery of the viral genome to the host chromatin as well as nuclear retention can be compromised during mitosis. Indeed, some viruses rely on a brief period during cellular division and nuclear envelope breakdown to access host chromatin (27). As the nuclear envelope disappears during cell mitosis, the viral genome also must be maintained in the nucleus. Chromatin tethering is then crucial to ensure delivery to and maintenance of the viral genome in the nucleus. Reviewing the virus families requiring a chromatin-tethering step, we can identify two strategies. The first one involves the interaction between viral components and cellular chromatin-binding factor. This way, the virus does not directly interact with chromatin but relies on cellular proteins that do so. Such a strategy has been described for viruses such as the gammaherpesviruses Epstein-Barr virus (EBV), Kaposi’s sarcoma-associated herpesvirus (KSHV), and papillomaviruses (11, 12). In the second strategy, viral proteins directly interact with the nucleosome. So far, retroviruses and DNA virus from the herpesviridae family have been shown to bind nucleosomes with determinants reminiscent of canonical cellular chromatin factors. Cryo-EM structure of the spumaretroviral prototype foamy virus intasome bound to the nucleosome highlighted the multivalency of the interaction with the nucleosome (9, 28). Other high-resolution structures of virus-nucleosome interaction involved short fragments of the viral proteins but captured the essence of the central role of arginine anchors and the acidic patch. In the following sections, we will focus on viral proteins that directly target the nucleosome acidic patch. We will describe the molecular mechanisms of viral protein tethering to host chromatin and the functional relevance during their respective replication cycle.

## KAPOSI’S SARCOMA-ASSOCIATED HERPESVIRUS LANA

**FIG 2:**
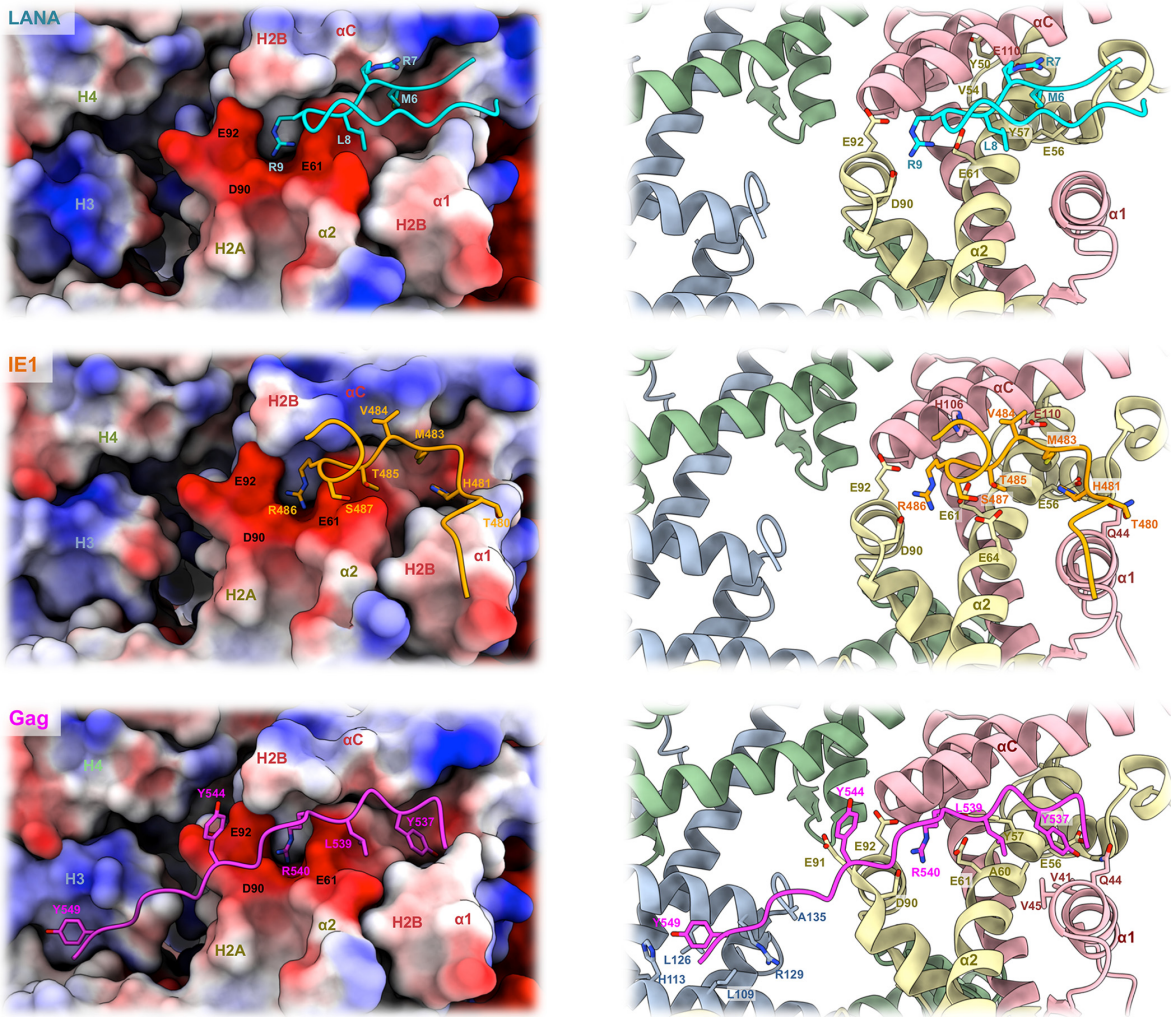
Detailed view of the interaction between H2A-H2B acidic patch and viral proteins. Histones are represented as electrostatic surface (left) or cartoon (right), with LANA peptide in cyan (PDB entry 1ZLA), IE1 peptide in orange (PDB entry 5E5A), and Gag peptide in magenta (PDVB entry 5MLU). Interacting residues are shown as sticks. Cartoon histones H2A, H2B, H3, and H4 are shown in yellow, red, blue, and green, respectively.

Human herpesvirus 8, also known as Kaposi’s sarcoma-associated herpesvirus, is the causative agent of Kaposi’s sarcoma, the predominant malignancy associated with AIDS but also primary effusion lymphoma and multicentric Castleman’s disease. Like all herpesviruses, KSHV can persist in a latent form in the infected cells (29). One main actor in the establishment of latency is LANA protein (30). LANA functions as a chromatin tether allowing retention and segregation of the viral genome during cell divisions. The protein also promotes viral DNA replication by recruiting cellular components of the replication machinery (31, 32). LANA-mediated chromatin binding of the KSHV genome is essential for virus survival (33). The first 22 amino acids of LANA contain the chromatin-tethering domain while the C-terminal domain mediates interaction with the tandem repeats of the viral genome (34, 35). Biochemical analysis suggested that the H2A-H2B dimer of the nucleosome is the target for LANA (36). In 2006, the structure of the first 23 residues of LANA bound to a nucleosome was solved using X-ray crystallography and revealed the molecular basis for LANA-nucleosome tethering (36). The peptide forms a hairpin structure and nicely accommodates the acidic patch, making several contacts with both H2A and H2B residues. Notably, the conserved arginine anchor 9 (R9) of LANA forms salt bridges with the acidic pocket made by E61, D90, and E92 of H2A (Fig. 2, top). The interaction is stabilized by additional salt bridges between LANA R7 and H2B alpha helix C (αC) residue E110 as well as hydrophobic contacts between LANA M6 and L8 with the hydrophobic pocket created along H2A α2 helix residues Y50, V54, Y57, and aliphatic portion of E56. Alanine substitutions within the first 13 amino acids abolish nucleosome binding, KSHV genome maintenance, and viral DNA replication, highlighting the central role of LANA in KSHV episome persistence during viral replication.

## HUMAN CYTOMEGALOVIRUS IE1 PROTEIN

The betaherpesvirus human cytomegalovirus is a widespread infectious agent causing serious pathologies in immunocompromised individuals, such as AIDS patients, organ transplant recipients, and children with primary and secondary immunodeficiencies (37, 38). The viral protein IE1 is a regulatory protein of 72 kDa expressed at the onset of infection whose functions have been associated with transcriptional regulation and innate immunity modulation (39, 40). Additionally, IE1 has been shown to associate with mitotic chromatin via a C-terminal chromatin-tethering domain (CTD; amino acids 476 to 491). The interaction involves H2A-H2B dimers and can be competed for by LANA, suggesting the same binding interface (14, 41). More recently, Fang et al. explored the structural basis for IE1 binding to the nucleosome core particle (14). Solved by X-ray crystallography, the structure of the IE1 chromatin tethering domain (amino acids 476 to 491) bound to a nucleosome shows that the viral protein occupies the acidic patch. The short peptide adopts a V-shaped conformation offering a network of interactions with H2A and H2B residues (Fig. 2, middle). Similar to LANA and many cellular chromatin factors, IE1 employs an arginine anchor, R486, to interact with the highly conserved acidic triad E61, D90, and E92 of H2A. Although distinctly folded, LANA and IE1-CTD share some common binding interfaces. Notably, similar to LANA M6, IE1 M483 is buried within the hydrophobic pocket formed between H2A α2 and histone H2B α1 and αC. The interaction is supported by hydrogen bonding between IE1 H481 and H2A residue E56 as well as hydrogen bonding between T485 and S487 with H2A E64. IE1 makes additional contacts with T480, interacting with H2B α1 helix Q44 and V484 binding with H2B αC H106 and E110. Amino acid substitutions in the IE1-nucleosome interface subsequently affect viral protein affinity for chromatin. However, the tethering domain of IE1 has been found to be dispensable during productive infection, raising the question of IE1-chromatin interaction function (41).

## SPUMARETROVIRUS GROUP ANTIGEN GAG PROTEIN

Historically called foamy viruses (FV) due to the foamy appearance of the infected cells, spumaretroviruses are retroviruses belonging to the distinct subfamily of *Spumaretrovirinae*. They are prevalent in several mammals, like nonhuman primates (*Prosimiispumavirus* and *Simiispumavirus*), felines (*Felispumavirus*), equines (*Equispumavirus*), and bovines (*Bovispumavirus*) (42). To this day, the physiopathology of this subfamily of retrovirus is understudied, yet infections appear apathogenic. Zoonotic transmissions between simiispumavirus and humans have been described and contributed to the isolation of the prototype foamy virus strain PFV. In addition to orthoretrovirinae human immunodeficiency virus 1 and 2 (HIV-1/2) and human T-lymphotropic virus 1 to 4 (HTLV1-4), PFV infections in humans arose from zoonotic transmission (43). Retroviral Gag proteins are encoded by the *gag* gene and constitute the main structural component of the viral particle. Gag is a polyprotein that will be matured by the retroviral protease. The cleaved protein products act at various steps during infection, such as intracellular trafficking, integration site selection, packaging, and viral assembly (44). Despite sharing some functional similarities, FV Gag proteins are quite divergent from their orthoretrovirinae homologs (45). Notably, the canonical nucleocapsid domain of the orthoretrovirus Gag C termini is absent from FV Gag. Instead, the protein harbors a disordered region containing stretches of glycine-arginine (GR) motifs. Pioneering works by Saib and Lindemann laboratories revealed the chromatin tethering ability of simiispumavirus PFV Gag protein (46, 47). The same observation was made more recently with felispumavirus Gag (48). As FVs only infect dividing cells, it was first suggested that chromatin tethering would promote nuclear translocation and retention of the viral particles in a manner similar to that of the DNA viruses described previously. Mutagenesis experiments isolated the chromatin-binding site (CBS) in the second GR motif of the PFV Gag C-terminal domain (46, 47). More recently, the crystal structure of PFV Gag CBS bound to a nucleosome was solved and shed light on the molecular mechanisms governing chromatin binding (49) (Fig. 2, bottom). The Gag CBS peptide adopts an extended conformation spanning the nucleosome side, contacting H2A, H2B, and both H3 chains. The H2A-H2B dimer contributes to most of the interface with Gag and hosts the canonical Gag arginine anchor R540, projecting into the acidic pocket made by H2A E61, D90, and E92. The interaction is supported by hydrophobic contacts between Gag Y537 and L539 with H2A α2 Y57 and A60 as well as H2B α1 V41 and V45. Further interactions involve hydrogen bonds between the hydroxyl group of Gag Y537 and the side chains of H2B Q44 and H2A E56 and between the main-chain carbonyl of Gag Y544 and the amide of H2A E91. As opposed to LANA and IE1, PFV Gag CBS reaches beyond the H2A-H2B acidic patch. Notably, Gag Y549 makes hydrophobic interactions with histone H3 L109, L126, and R129 as well as hydrogen bonds with H113 and the main-chain carboxylate of A135.

In contrast to herpesviridae that maintain viral genomes as episomes, retroviruses integrate their viral DNA into host chromatin (50). Several studies showed that selection of the integration site into the host chromatin is not random. Instead, integration displays retroviral genus-specific genomic preferences. The molecular mechanisms responsible for integration site selection are not fully described, but major determinants point to a critical role of interaction between retroviral integrase and Gag products with specific cellular proteins (51). For example, in the case of the lentivirus HIV-1 the interaction between the viral capsid (a Gag product) and the cellular protein cleavage and polyadenylation specificity factor subunit 6 (CPSF6) plays a central role in targeting the integration into gene-dense regions (52). Subsequent layers of integration site selections imply the interaction between the HIV-1 integrase with lens epithelium-derived growth factor protein (LEDGF/p75) as well as nucleosome components (53–55). The mechanisms governing FV integration site selection are still obscure. However, abolishment of the chromatin binding ability of PFV Gag protein induces a dramatic redistribution of integration sites, indicating a central role of Gag-chromatin interaction in FV integration site selection (49).

## MURINE LEUKEMIA VIRUS P12 PROTEIN

Murine leukemia virus (MLV) is a retrovirus of the orthoretrovirinae subfamily and belongs to the gammaretrovirus genus. MLVs are among the simplest retroviruses, encoding only three polyproteins (Gag, Pol, and Env) used for the production of progeny particles. Although infection by MLV does not show obvious physiological effects on cells, infected hosts develop tumors with long latency periods (56). Transformation of cells was shown to be the result of the activation of cellular proto-oncogenes following integration (insertional mutagenesis). The study of MLVs has provided many insights on cancer biology, retrovirology and for the development of medical tools like gene therapy vectors.

Like FVs, gammaretroviruses are dependent on nuclear envelope breakdown during mitosis for gaining access to host chromatin. An early study identified a role of the gag cleavage product, p12, in nuclear retention of MLV DNA (57). Additional characterization studies of MLV preintegration complexes (PICs) confirmed the role of p12 in tethering host chromatin and revealed its importance in both early and late stages of viral replication (58, 59). p12 is composed of two functional domains, the N-terminal domain, which binds and stabilizes the capsid lattice, and the C-terminal domain, responsible for chromatin tethering (60). Chromatin capture by MLV p12 protein seems to be finely regulated, as several works pointed to a role of p12 phosphorylation in modulating chromatin affinity (61–63). To this day, the exact functions of this posttranslational modification remain elusive. However, alteration of p12’s ability to interact with chromatin by mutating the C-terminal domain shows profound deleterious effects on infection. These defective viruses can be partially rescued by complementation with a heterologous chromatin-binding motif like LANA or PFV CBS (64).

The exact structural mechanisms underlying nucleosome interaction by viral p12 are still not fully understood. However, recent biophysical approaches show that p12 binding to chromatin assembled *in vitro* can be competed for by PFV Gag CBS but not by chromatin-binding deficient substitution R540Q, suggesting that p12 binds directly to the nucleosome and targets the H2A-H2B acidic patch (63). Future work on the structural basis for p12 binding to the nucleosome will be of great interest to dissect the exact molecular details of p12-chromatin interactions and the role of p12 phosphorylation.

## THE NEXUS BETWEEN VIRAL PROTEIN TETHERING TO NUCLEOSOME AND ALTERATION OF CHROMATIN STRUCTURE

As described in the previous section, some viruses require a tight entanglement with host chromatin during specific steps of their replication cycle. These examples are probably just the tip of the iceberg, as transactions in the host cell nucleus are a hallmark of many more viruses. Increasing knowledge on virus biology and evolution showed that they are master manipulators of the infected cells. This requires an extreme condensation of functions into the smallest possible unit. This is exemplified by overlapping gene sequences, host machinery hijacking, and multifunctional viral proteins. As mentioned above, the H2A-H2B acidic patch has been implicated in mediating nucleosome-nucleosome contacts via interaction with the H4 N-terminal tail. Since viral proteins occupy the same interface, it is conceivable that their binding prevents or modulates nucleosome-nucleosome interactions. However, such interference might require a significant amount of nuclear protein to shift the binding equilibrium. Interestingly, LANA, IE1, and the retroviral Gag products all traffic inside the nucleus and are among the most abundant proteins expressed during respective viral replication (65–67). The quantity of incoming viral proteins required to affect the global chromatin architecture is currently unknown; however, local alterations by a minimal amount of viral proteins can be of biological relevance during specific replication steps.

In the following section, we will examine the available facts showing that acidic patch occupancy by viral proteins can interfere with higher-order chromatin structures and then discuss the opening questions.

First, using biochemical experiments, Luger and colleagues showed that KHSV LANA could promote nucleosome-nucleosome interactions and chromatin oligomerization *in vitro* (68). By measuring the ability of a chromatin array to oligomerize as a function of divalent cation MgCl_2_ concentration, they monitored the effect of LANA peptides. Wild-type LANA peptide could promote chromatin self-association, whereas mutant LANA peptide unable to bind the nucleosome showed no effect. This effect seems independent of H4 tail, as a similar phenotype was observed on chromatin arrays assembled without H4 tails. The author hypothesized that LANA-mediated chromatin compaction acts through quenching specific charged surfaces on the histone core, promoting oligonucleosome formation. Indeed, by mutating the charged residues of H2A that contribute to LANA binding, they could partly recapitulate the effect of LANA addition on wild-type arrays. The functional relevance of this phenotype was also investigated *in cellulo*. LANA peptides fused to green fluorescent protein were expressed in U2OS cells, and the integrity of chromatin was monitored by Hoechst 33258 staining. As opposed to nucleosome binding-deficient LANA peptides, wild-type LANA expression induces large regions of Hoechst exclusion, suggesting an alteration of the chromatin structure. Of note, similar results were obtained with a short peptide derived from interleukin-33 (IL-33) which chromatin-binding motif shares striking similarity with LANA. (69).

It was then suggested that the capacity of an array of nucleosomes to self-associate results from the balance between attractive and repulsive domains on the histone cores. Such balance can be modulated by differential acidic patch binding, as seen with LANA and H4 tail.

**FIG 3:**
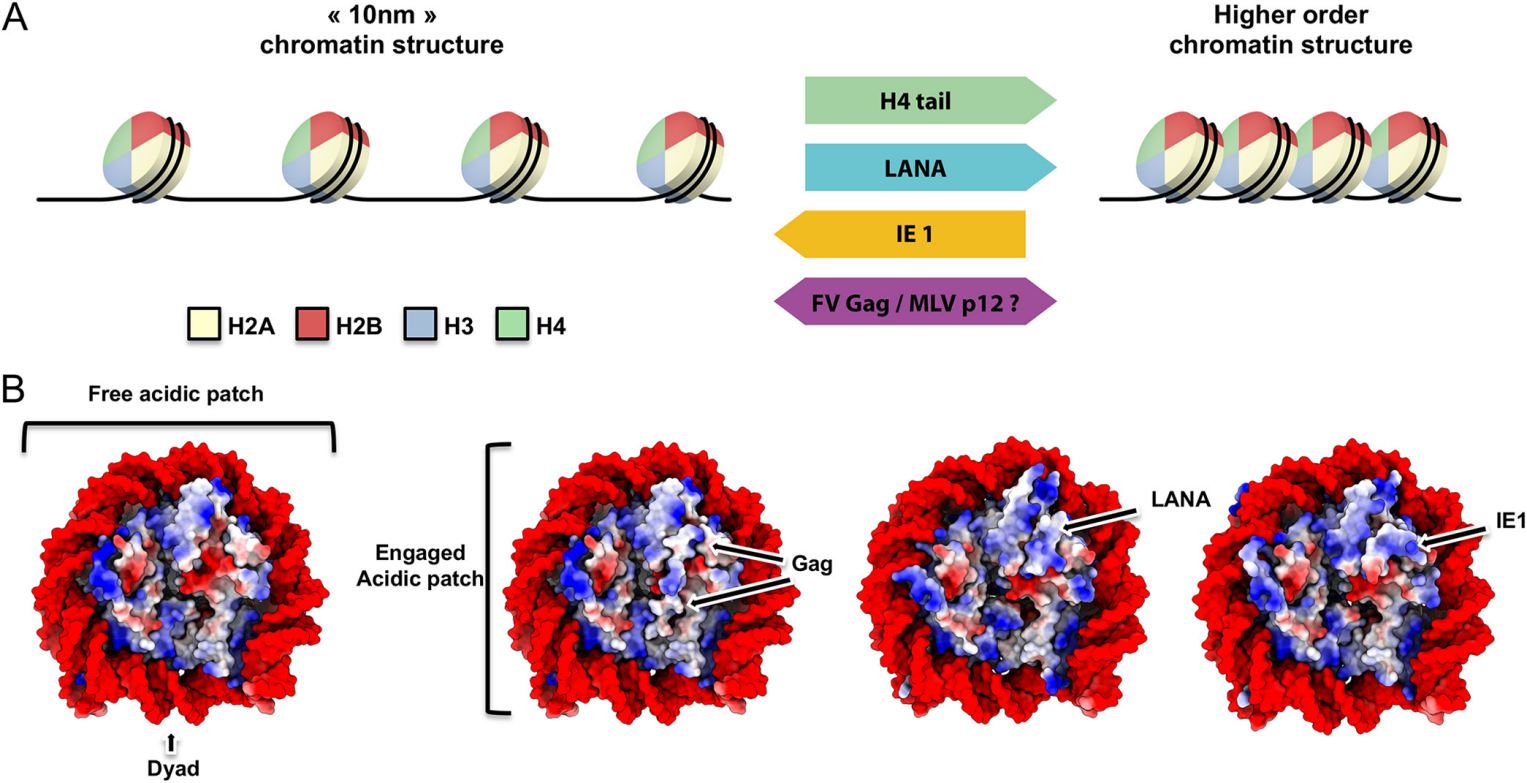
Chromatin structure alteration by viral protein tethers. (A) H4 tail-acidic patch competition model. Transitions between a 10-nm chromatin structure and higher-order chromatin structure involve binding of H4 tail to neighboring nucleosome acidic patches. This interaction can be competed for by viral proteins interacting with H2A-H2B acidic patch, subsequently shifting the equilibrium state toward a more open or folded chromatin. (B) Electrostatic surface representation of the nucleosome engaged by viral proteins (indicated by an arrow).

With the recent breakthroughs on *in vitro* chromatin fiber assembly and structure, Li and colleagues investigated further the interference induced by viral acidic patch binders (14). Using analytical ultracentrifugation in sedimentation velocity analyses, they compared the effect of IE1 and LANA on both 10-nm and 30-nm chromatin fibers assembled *in vitro*. While the 10-nm fiber sedimentation was unaffected by IE1, the viral protein induced a lower sedimentation coefficient of the 30-nm fiber, consistent with decondensation of the assembled chromatin. Quantitative measurements showed that the altered folding state of the chromatin array in the presence of IE1 constitutes a unique remodeled structure, resembling neither 10-nm nor 30-nm fiber. Conversely, LANA does not affect the folding of the 30-nm fiber but, consistent with the previously published observations, induces a condensation of the 10-nm array. The opposite effect of these two acidic patch binders suggests that distinct contacts made within the acidic patch could influence the outcome of chromatin folding modulation. These first reports of chromatin remodeling by viral proteins point to a complex role of the acidic patch in the mechanics of higher-order chromatin folding. It was then suggested that despite the common arginine anchor motif interacting with H2A acidic triad, the differential binding interface between viral proteins and core histones generates various occluded and accessible zones that, in turn, regulate the chromatin-folding capacity (Fig. 3A). The question of whether other viral proteins like PFV Gag and MLV p12 can exert the same remodeling action on chromatin templates is still open.

Besides physical accessibility and competition with H4 tail, binding of viral proteins can change the electrostatic potential of the nucleosome surface, creating an additional layer of potential regulation. Analysis of the charge distribution shows that the presence of viral proteins can selectively quench or invert charges on the histone core surface (Fig. 3B). For example, PFV Gag makes extensive contacts with the histone core and almost completely masks the H2A-H2B acidic patch, neutralizing the surface charges. LANA presence masks and inverts the exposed charges, while IE1 exposes both basic and acidic surfaces. These modifications can be detrimental for long-range chromatin interactions (70) and may underline virus-specific chromatin alteration beyond the short-range H4 tail-acidic patch interaction. Additionally, changes in the electrostatic potential may alter the recruitment of chromatin factors that bind away from the acidic patch. Many of these factors are DNA binding, chromatin remodeling, or modifying complexes. These complexes are regulators of the chromatin architecture and are key in processes like DNA transcription, replication, and repair (9, 71). Failure in their chromatin interaction has been linked to diseases and cancers (72).

It is tempting to speculate that chromatin architecture modulation by the presence of viral proteins can have profound physiological expressions, like alteration of cellular gene expression, spreading of modified chromatin state, or even generation of phase-separated chromatin domains (73). Such virally induced chromatin territories could result in the creation of optimal viral replications centers (or viral factories) within the nucleus. Although more examples of higher-order chromatin structure alteration *in vitro* by viral proteins will be of great interest, the field now needs to generate assays aiming at unraveling the mechanisms involved during chromatin tethering by viral proteins at the cellular level.

## CONCLUSION

Over the past 5 years, the abundance of high-resolution structures of nucleosomes in complex with chromatin factors has provided great insights into the molecular mechanisms governing nucleosome capture. Tethering of proteins to chromatin is fundamental in the maintenance of genome integrity and cellular homeostasis. The H2A-H2B acidic patch is a hot spot for nucleosome recognition by chromatin factors containing a shared motif of interaction called an arginine anchor. Additionally, evidence shows that interactions between histone H4 tail and the H2A-H2B acidic patch is a central determinant for short- and long-range chromatin structure organization.

Nucleosome capture is a hallmark of several viral proteins. Careful examination of the binding interface revealed a shared mechanism of interaction to the nucleosome acidic patch via an arginine anchor. Initially believed to be only involved in nuclear retention, several studies point to a more complex role in various replication steps.

Disruption of the H4 tail-acidic patch interaction by the presence of non-self viral proteins has been shown to cause interference in chromatin folding *in vitro*. Future research will be of great interest to characterize further the interplay between viruses and host chromatin. Expanding the repertoire of known virus-nucleosome tethers as well as unraveling the cellular consequences of their acidic patch occupancy will be key in a better comprehension of virus evolution and chromatin biology while opening a potential new area of antiviral strategies.

## References

[1] Luger K, Mader AW, Richmond RK, Sargent DF, Richmond TJ. 1997. Crystal structure of the nucleosome core particle at 2.8 A resolution. Nature 389:251–260. doi:10.1038/38444. 9305837

[2] Ehrenhofer-Murray AE. 2004. Chromatin dynamics at DNA replication, transcription and repair. Eur J Biochem 271:2335–2349. doi:10.1111/j.1432-1033.2004.04162.x. 15182349

[3] Chen P, Li W, Li G. 2021. Structures and functions of chromatin fibers. Annu Rev Biophys 50:95–116. doi:10.1146/annurev-biophys-062920-063639. 33957053

[4] Dorigo B, Schalch T, Bystricky K, Richmond TJ. 2003. Chromatin fiber folding: requirement for the histone H4 N-terminal tail. J Mol Biol 327:85–96. doi:10.1016/S0022-2836(03)00025-1. 12614610

[5] McGinty RK, Tan S. 2015. Nucleosome structure and function. Chem Rev 115:2255–2273. doi:10.1021/cr500373h. 25495456 PMC4378457

[6] McBride MJ, Mashtalir N, Winter EB, Dao HT, Filipovski M, D'Avino AR, Seo H-S, Umbreit NT, St Pierre R, Valencia AM, Qian K, Zullow HJ, Jaffe JD, Dhe-Paganon S, Muir TW, Kadoch C. 2020. The nucleosome acidic patch and H2A ubiquitination underlie mSWI/SNF recruitment in synovial sarcoma. Nat Struct Mol Biol 27:836–845. doi:10.1038/s41594-020-0466-9. 32747783 PMC7714695

[7] Valencia AM, Collings CK, Dao HT, St Pierre R, Cheng Y-C, Huang J, Sun Z-Y, Seo H-S, Mashtalir N, Comstock DE, Bolonduro O, Vangos NE, Yeoh ZC, Dornon MK, Hermawan C, Barrett L, Dhe-Paganon S, Woolf CJ, Muir TW, Kadoch C. 2019. Recurrent SMARCB1 mutations reveal a nucleosome acidic patch interaction site that potentiates mSWI/SNF complex chromatin remodeling. Cell 179:1342–1356. doi:10.1016/j.cell.2019.10.044. 31759698 PMC7175411

[8] Zhou K, Gaullier G, Luger K. 2019. Nucleosome structure and dynamics are coming of age. Nat Struct Mol Biol 26:3–13. doi:10.1038/s41594-018-0166-x. 30532059 PMC7386248

[9] McGinty RK, Tan S. 2021. Principles of nucleosome recognition by chromatin factors and enzymes. Curr Opin Struct Biol 71:16–26. doi:10.1016/j.sbi.2021.05.006. 34198054 PMC8648869

[10] Lucic B, de Castro IJ, Lusic M. 2021. Viruses in the nucleus. Cold Spring Harb Perspect Biol 13:a039446. doi:10.1101/cshperspect.a039446. 33753405 PMC8327829

[11] Aydin I, Schelhaas M. 2016. Viral genome tethering to host cell chromatin: cause and consequences. Traffic 17:327–340. doi:10.1111/tra.12378. 26787361

[12] Delelis O, Zamborlini A, Thierry S, Saïb A. 2010. Chromosomal tethering and proviral integration. Biochim Biophys Acta 1799:207–216. doi:10.1016/j.bbagrm.2009.08.005. 19683084

[13] Kale S, Goncearenco A, Markov Y, Landsman D, Panchenko AR. 2019. Molecular recognition of nucleosomes by binding partners. Curr Opin Struct Biol 56:164–170. doi:10.1016/j.sbi.2019.03.010. 30991239 PMC6656623

[14] Fang Q, Chen P, Wang M, Fang J, Yang N, Li G, Xu R-M. 2016. Human cytomegalovirus IE1 protein alters the higher-order chromatin structure by targeting the acidic patch of the nucleosome. Elife 5:e11911. doi:10.7554/eLife.11911. 26812545 PMC4764553

[15] Skrajna A, Goldfarb D, Kedziora KM, Cousins EM, Grant GD, Spangler CJ, Barbour EH, Yan X, Hathaway NA, Brown NG, Cook JG, Major MB, McGinty RK. 2020. Comprehensive nucleosome interactome screen establishes fundamental principles of nucleosome binding. Nucleic Acids Res 48:9415–9432. doi:10.1093/nar/gkaa544. 32658293 PMC7515726

[16] Lobbia VR, Trueba Sanchez MC, van Ingen H. 2021. Beyond the nucleosome: nucleosome-protein interactions and higher order chromatin structure. J Mol Biol 433:166827. doi:10.1016/j.jmb.2021.166827. 33460684

[17] Wakamori M, Fujii Y, Suka N, Shirouzu M, Sakamoto K, Umehara T, Yokoyama S. 2015. Intra- and inter-nucleosomal interactions of the histone H4 tail revealed with a human nucleosome core particle with genetically-incorporated H4 tetra-acetylation. Sci Rep 5:17204. doi:10.1038/srep17204. 26607036 PMC4660432

[18] Chen Q, Yang R, Korolev N, Liu CF, Nordenskiöld L. 2017. Regulation of nucleosome stacking and chromatin compaction by the histone H4 N-terminal tail–H2A acidic patch interaction. J Mol Biol 429:2075–2092. doi:10.1016/j.jmb.2017.03.016. 28322915

[19] Song F, Chen P, Sun D, Wang M, Dong L, Liang D, Xu R-M, Zhu P, Li G. 2014. Cryo-EM study of the chromatin fiber reveals a double helix twisted by tetranucleosomal units. Science 344:376–380. doi:10.1126/science.1251413. 24763583

[20] Maeshima K, Ide S, Babokhov M. 2019. Dynamic chromatin organization without the 30-nm fiber. Curr Opin Cell Biol 58:95–104. doi:10.1016/j.ceb.2019.02.003. 30908980

[21] Ou HD, Phan S, Deerinck TJ, Thor A, Ellisman MH, O’Shea CC. 2017. ChromEMT: visualizing 3D chromatin structure and compaction in interphase and mitotic cells. Science 357:eaag0025. doi:10.1126/science.aag0025. 28751582 PMC5646685

[22] Xu P, Mahamid J, Dombrowski M, Baumeister W, Olins AL, Olins DE. 2021. Interphase epichromatin: last refuge for the 30-nm chromatin fiber? Chromosoma 130:91–102. doi:10.1007/s00412-021-00759-8. 34091761

[23] Baldi S, Korber P, Becker PB. 2020. Beads on a string-nucleosome array arrangements and folding of the chromatin fiber. Nat Struct Mol Biol 27:109–118. doi:10.1038/s41594-019-0368-x. 32042149

[24] Kan P-Y, Caterino TL, Hayes JJ. 2009. The H4 tail domain participates in intra- and internucleosome interactions with protein and DNA during folding and oligomerization of nucleosome arrays. Mol Cell Biol 29:538–546. doi:10.1128/MCB.01343-08. 19001093 PMC2612503

[25] Sinha D, Shogren-Knaak MA. 2010. Role of direct interactions between the histone H4 tail and the H2A core in long range nucleosome contacts. J Biol Chem 285:16572–16581. doi:10.1074/jbc.M109.091298. 20351095 PMC2878085

[26] Dodonova SO, Zhu F, Dienemann C, Taipale J, Cramer P. 2020. Nucleosome-bound SOX2 and SOX11 structures elucidate pioneer factor function. Nature 580:669–672. doi:10.1038/s41586-020-2195-y. 32350470

[27] Fay N, Panté N. 2015. Nuclear entry of DNA viruses. Front Microbiol 6:467. doi:10.3389/fmicb.2015.00467. 26029198 PMC4429625

[28] Maskell DP, Renault L, Serrao E, Lesbats P, Matadeen R, Hare S, Lindemann D, Engelman AN, Costa A, Cherepanov P. 2015. Structural basis for retroviral integration into nucleosomes. Nature 523:366–369. [PMC] doi:10.1038/nature14495. 26061770 PMC4530500

[29] De Leo A, Calderon A, Lieberman PM. 2020. Control of viral latency by episome maintenance proteins. Trends Microbiol 28:150–162. doi:10.1016/j.tim.2019.09.002. 31624007 PMC6980450

[30] Godfrey A, Anderson J, Papanastasiou A, Takeuchi Y, Boshoff C. 2005. Inhibiting primary effusion lymphoma by lentiviral vectors encoding short hairpin RNA. Blood 105:2510–2518. doi:10.1182/blood-2004-08-3052. 15572586

[31] Verma SC, Choudhuri T, Kaul R, Robertson ES. 2006. Latency-associated nuclear antigen (LANA) of Kaposi’s sarcoma-associated herpesvirus interacts with origin recognition complexes at the LANA binding sequence within the terminal repeats. J Virol 80:2243–2256. doi:10.1128/JVI.80.5.2243-2256.2006. 16474132 PMC1395374

[32] Sun Q, Tsurimoto T, Juillard F, Li L, Li S, De León Vázquez E, Chen S, Kaye K. 2014. Kaposi’s sarcoma-associated herpesvirus LANA recruits the DNA polymerase clamp loader to mediate efficient replication and virus persistence. Proc Natl Acad Sci USA 111:11816–11821. doi:10.1073/pnas.1404219111. 25071216 PMC4136584

[33] Ballestas ME, Chatis PA, Kaye KM. 1999. Efficient persistence of extrachromosomal KSHV DNA mediated by latency-associated nuclear antigen. Science 284:641–644. doi:10.1126/science.284.5414.641. 10213686

[34] Piolot T, Tramier M, Coppey M, Nicolas J-C, Marechal V. 2001. Close but distinct regions of human herpesvirus 8 latency-associated nuclear antigen 1 are responsible for nuclear targeting and binding to human mitotic chromosomes. J Virol 75:3948–3959. doi:10.1128/JVI.75.8.3948-3959.2001. 11264383 PMC114885

[35] Barbera AJ, Ballestas ME, Kaye KM. 2004. The Kaposi’s sarcoma-associated herpesvirus latency-associated nuclear antigen 1 N terminus is essential for chromosome association, DNA replication, and episome persistence. J Virol 78:294–301. doi:10.1128/jvi.78.1.294-301.2004. 14671111 PMC303411

[36] Barbera AJ, Chodaparambil JV, Kelley-Clarke B, Joukov V, Walter JC, Luger K, Kaye KM. 2006. The nucleosomal surface as a docking station for Kaposi’s sarcoma herpesvirus LANA. Science 311:856–861. doi:10.1126/science.1120541. 16469929

[37] Griffiths P, Reeves M. 2021. Pathogenesis of human cytomegalovirus in the immunocompromised host. Nat Rev Microbiol 19:759–773. doi:10.1038/s41579-021-00582-z. 34168328 PMC8223196

[38] Bateman CM, Kesson A, Powys M, Wong M, Blyth E. 2021. Cytomegalovirus infections in children with primary and secondary immune deficiencies. Viruses 13:2001. doi:10.3390/v13102001. 34696432 PMC8538792

[39] Pignoloni B, Fionda C, Dell'Oste V, Luganini A, Cippitelli M, Zingoni A, Landolfo S, Gribaudo G, Santoni A, Cerboni C. 2016. Distinct roles for human cytomegalovirus immediate early proteins IE1 and IE2 in the transcriptional regulation of MICA and PVR/CD155 expression. J Immunol 197:4066–4078. doi:10.4049/jimmunol.1502527. 27733551

[40] Dell'Oste V, Biolatti M, Galitska G, Griffante G, Gugliesi F, Pasquero S, Zingoni A, Cerboni C, De Andrea M. 2020. Tuning the orchestra: HCMV vs. innate immunity. Front Microbiol 11:661. doi:10.3389/fmicb.2020.00661. 32351486 PMC7174589

[41] Reinhardt J, Smith GB, Himmelheber CT, Azizkhan-Clifford J, Mocarski ES. 2005. The carboxyl-terminal region of human cytomegalovirus IE1491aa contains an acidic domain that plays a regulatory role and a chromatin-tethering domain that is dispensable during viral replication. J Virol 79:225–233. doi:10.1128/JVI.79.1.225-233.2005. 15596818 PMC538725

[42] Rethwilm A, Bodem J. 2013. Evolution of foamy viruses: the most ancient of all retroviruses. Viruses 5:2349–2374. doi:10.3390/v5102349. 24072062 PMC3814592

[43] Pinto-Santini DM, Stenbak CR, Linial ML. 2017. Foamy virus zoonotic infections. Retrovirology 14:55. doi:10.1186/s12977-017-0379-9. 29197389 PMC5712078

[44] Bell NM, Lever AML. 2013. HIV Gag polyprotein: processing and early viral particle assembly. Trends Microbiol 21:136–144. doi:10.1016/j.tim.2012.11.006. 23266279

[45] Müllers E. 2013. The foamy virus Gag proteins: what makes them different? Viruses 5:1023–1041. doi:10.3390/v5041023. 23531622 PMC3705263

[46] Tobaly-Tapiero J, Bittoun P, Lehmann-Che J, Delelis O, Giron M-L, de Thé H, Saïb A. 2008. Chromatin tethering of incoming foamy virus by the structural Gag protein. Traffic 9:1717–1727. doi:10.1111/j.1600-0854.2008.00792.x. 18627573

[47] Müllers E, Stirnnagel K, Kaulfuss S, Lindemann D. 2011. Prototype foamy virus gag nuclear localization: a novel pathway among retroviruses. J Virol 85:9276–9285. doi:10.1128/JVI.00663-11. 21715475 PMC3165767

[48] Wei G, Kehl T, Bao Q, Benner A, Lei J, Löchelt M. 2018. The chromatin binding domain, including the QPQRYG motif, of feline foamy virus Gag is required for viral DNA integration and nuclear accumulation of Gag and the viral genome. Virology 524:56–68. doi:10.1016/j.virol.2018.08.007. 30145377

[49] Lesbats P, Serrao E, Maskell DP, Pye VE, O’Reilly N, Lindemann D, Engelman AN, Cherepanov P. 2017. Structural basis for spumavirus GAG tethering to chromatin. Proc Natl Acad Sci USA 114:5509–5514. doi:10.1073/pnas.1621159114. 28490494 PMC5448199

[50] Lesbats P, Engelman AN, Cherepanov P. 2016. Retroviral DNA integration. Chem Rev 116:12730–12757. doi:10.1021/acs.chemrev.6b00125. 27198982 PMC5084067

[51] Engelman AN. 2021. HIV capsid and integration targeting. Viruses 13:125. doi:10.3390/v13010125. 33477441 PMC7830116

[52] Sowd GA, Serrao E, Wang H, Wang W, Fadel HJ, Poeschla EM, Engelman AN. 2016. A critical role for alternative polyadenylation factor CPSF6 in targeting HIV-1 integration to transcriptionally active chromatin. Proc Natl Acad Sci USA 113:E1054–E1063. doi:10.1073/pnas.1524213113. 26858452 PMC4776470

[53] Wang H, Jurado KA, Wu X, Shun M-C, Li X, Ferris AL, Smith SJ, Patel PA, Fuchs JR, Cherepanov P, Kvaratskhelia M, Hughes SH, Engelman A. 2012. HRP2 determines the efficiency and specificity of HIV-1 integration in LEDGF/p75 knockout cells but does not contribute to the antiviral activity of a potent LEDGF/p75-binding site integrase inhibitor. Nucleic Acids Res 40:11518–11530. doi:10.1093/nar/gks913. 23042676 PMC3526291

[54] Serrao E, Krishnan L, Shun M-C, Li X, Cherepanov P, Engelman A, Maertens GN. 2014. Integrase residues that determine nucleotide preferences at sites of HIV-1 integration: implications for the mechanism of target DNA binding. Nucleic Acids Res 42:5164–5176. doi:10.1093/nar/gku136. 24520116 PMC4005685

[55] Benleulmi MS, Matysiak J, Robert X, Miskey C, Mauro E, Lapaillerie D, Lesbats P, Chaignepain S, Henriquez DR, Calmels C, Oladosu O, Thierry E, Leon O, Lavigne M, Andreola M-L, Delelis O, Ivics Z, Ruff M, Gouet P, Parissi V. 2017. Modulation of the functional association between the HIV-1 intasome and the nucleosome by histone amino-terminal tails. Retrovirology 14:54. doi:10.1186/s12977-017-0378-x. 29179726 PMC5704366

[56] Rein A. 2011. Murine leukemia viruses: objects and organisms. Adv Virol 2011:403419. doi:10.1155/2011/403419. 22312342 PMC3265304

[57] Yuan B, Li X, Goff SP. 1999. Mutations altering the moloney murine leukemia virus p12 Gag protein affect virion production and early events of the virus life cycle. EMBO J 18:4700–4710. doi:10.1093/emboj/18.17.4700. 10469649 PMC1171543

[58] Prizan-Ravid A, Elis E, Laham-Karam N, Selig S, Ehrlich M, Bacharach E. 2010. The Gag cleavage product, p12, is a functional constituent of the murine leukemia virus pre-integration complex. PLoS Pathog 6:e1001183. doi:10.1371/journal.ppat.1001183. 21085616 PMC2978732

[59] Elis E, Ehrlich M, Prizan-Ravid A, Laham-Karam N, Bacharach E. 2012. p12 tethers the murine leukemia virus pre-integration complex to mitotic chromosomes. PLoS Pathog 8:e1003103. doi:10.1371/journal.ppat.1003103. 23300449 PMC3531515

[60] Wight DJ, Boucherit VC, Nader M, Allen DJ, Taylor IA, Bishop KN. 2012. The gammaretroviral p12 protein has multiple domains that function during the early stages of replication. Retrovirology 9:83. doi:10.1186/1742-4690-9-83. 23035841 PMC3492146

[61] Brzezinski JD, Felkner R, Modi A, Liu M, Roth MJ. 2016. Phosphorylation requirement of murine leukemia virus p12. J Virol 90:11208–11219. doi:10.1128/JVI.01178-16. 27707931 PMC5126377

[62] Brzezinski JD, Modi A, Liu M, Roth MJ. 2016. Repression of the chromatin-tethering domain of murine leukemia virus p12. J Virol 90:11197–11207. doi:10.1128/JVI.01084-16. 27707926 PMC5126376

[63] Wanaguru M, Barry DJ, Benton DJ, O'Reilly NJ, Bishop KN. 2018. Murine leukemia virus p12 tethers the capsid-containing pre-integration complex to chromatin by binding directly to host nucleosomes in mitosis. PLoS Pathog 14:e1007117. doi:10.1371/journal.ppat.1007117. 29906285 PMC6021111

[64] Schneider WM, Brzezinski JD, Aiyer S, Malani N, Gyuricza M, Bushman FD, Roth MJ. 2013. Viral DNA tethering domains complement replication-defective mutations in the p12 protein of MuLV Gag. Proc Natl Acad Sci USA 110:9487–9492. doi:10.1073/pnas.1221736110. 23661057 PMC3677494

[65] Jeong JH, Orvis J, Kim JW, McMurtrey CP, Renne R, Dittmer DP. 2004. Regulation and autoregulation of the promoter for the latency-associated nuclear antigen of Kaposi’s Sarcoma-associated herpesvirus. J Biol Chem 279:16822–16831. doi:10.1074/jbc.M312801200. 14742422

[66] Nevels M, Paulus C, Shenk T. 2004. Human cytomegalovirus immediate-early 1 protein facilitates viral replication by antagonizing histone deacetylation. Proc Natl Acad Sci USA 101:17234–17239. doi:10.1073/pnas.0407933101. 15572445 PMC535392

[67] Swiersy A, Wiek C, Reh J, Zentgraf H, Lindemann D. 2011. Orthoretroviral-like prototype foamy virus gag-pol expression is compatible with viral replication. Retrovirology 8:66. doi:10.1186/1742-4690-8-66. 21843316 PMC3196705

[68] Chodaparambil JV, Barbera AJ, Lu X, Kaye KM, Hansen JC, Luger K. 2007. A charged and contoured surface on the nucleosome regulates chromatin compaction. Nat Struct Mol Biol 14:1105–1107. doi:10.1038/nsmb1334. 17965723 PMC2366819

[69] Roussel L, Erard M, Cayrol C, Girard J-P. 2008. Molecular mimicry between IL-33 and KSHV for attachment to chromatin through the H2A-H2B acidic pocket. EMBO Rep 9:1006–1012. doi:10.1038/embor.2008.145. 18688256 PMC2572127

[70] Zhou H-X, Pang X. 2018. Electrostatic interactions in protein structure, folding, binding, and condensation. Chem Rev 118:1691–1741. doi:10.1021/acs.chemrev.7b00305. 29319301 PMC5831536

[71] Zhou K, Gaullier G, Luger K. 2019. Nucleosome structure and dynamics are coming of age. Nat Struct Mol Biol 26:3–13. doi:10.1038/s41594-018-0166-x. 30532059 PMC7386248

[72] Mirabella AC, Foster BM, Bartke T. 2016. Chromatin deregulation in disease. Chromosoma 125:75–93. doi:10.1007/s00412-015-0530-0. 26188466 PMC4761009

[73] Lopez N, Camporeale G, Salgueiro M, Borkosky SS, Visentín A, Peralta-Martinez R, Loureiro ME, de Prat-Gay G. 2021. Deconstructing virus condensation. PLoS Pathog 17:e1009926. doi:10.1371/journal.ppat.1009926. 34648608 PMC8516229

[74] Pettersen EF, Goddard TD, Huang CC, Meng EC, Couch GS, Croll TI, Morris JH, Ferrin TE. 2021. UCSF ChimeraX: structure visualization for researchers, educators, and developers. Protein Sci 30:70–82. doi:10.1002/pro.3943. 32881101 PMC7737788

